# Substrate Integrated Waveguide on Glass with Vacuum-Filled Tin Through Glass Vias for Millimeter-Wave Applications

**DOI:** 10.3390/mi16010012

**Published:** 2024-12-26

**Authors:** Seung-Han Chung, Ho-Sun Yeom, Che-Heung Kim, Yong-Kweon Kim, Seung-Ki Lee, Chang-Wook Baek, Jae-Hyoung Park

**Affiliations:** 1 Department of Electrical and Computer Engineering, Seoul National University, 1 Gwanak-ro, Gwanak-gu, Seoul 08826, Republic of Korea; jsh9105@snu.ac.kr (S.-H.C.); wollmoon@snu.ac.kr (C.-H.K.); yongkkim@snu.ac.kr (Y.-K.K.); 2 Department of Foundry Engineering, Dankook University, Yongin 16890, Republic of Korea; yhs5659@naver.com; 3 Department of Semiconductor Convergence Engineering, Dankook University, Yongin 16890, Republic of Korea; skilee@dankook.ac.kr; 4 School of Electrical and Electronics Engineering, Chung-Ang University, 84 Heukseok-ro, Dongjak-gu, Seoul 06974, Republic of Korea

**Keywords:** substrate integrated waveguide (SIW), through glass via (TGV), tin (Sn) vias, vacuum suctioning system, micromachining

## Abstract

This paper presents a novel approach to fabricate substrate integrated waveguides (SIWs) on glass substrates with tin (Sn) through glass vias (TGVs) tailored for millimeter-wave applications. The fabrication process employs a custom-designed vacuum suctioning system to rapidly fill precise TGV holes in the glass substrate, which are formed by wafer-level glass reflow micromachining techniques with molten tin in a minute. This method offers a very fast and cost-effective alternative for complete via filling without voids compared to the conventional metallization techniques such as electroplating or sputtering. An SIW with a 3-dB cutoff frequency of 17.2 GHz was fabricated using the proposed process. The fabricated SIW shows an average insertion loss of 1.65 ± 0.54 dB across the 20–35 GHz range. These results highlight the potential of glass substrates with tin TGVs for fabricating millimeter-wave devices.

## 1. Introduction

Recent progress in autonomous driving and artificial intelligence (AI) has led to a growing demand for millimeter-wave devices, driven by the need for high bandwidth, low latency, and minimal signal loss [1]. In millimeter-wave systems, components such as processors, antennas, filters, and amplifiers are integrated onto a single board, requiring compact and efficient design approaches. Traditionally, microstrip lines and coplanar waveguides (CPWs) have been employed for signal transmission between these components. However, at frequencies exceeding 20 GHz, conventional transmission lines exhibit higher losses, rendering them unsuitable for millimeter-wave applications. To overcome these limitations, substrate integrated waveguides (SIWs), inspired by rectangular waveguides, have been proposed and widely adopted for various applications such as filters, antennas and other passive devices [2,3,4,5,6,7,8,9,10]. SIWs use conductive through-substrate vias to connect the top transmission line to the bottom ground plane, effectively replacing the metallic sidewalls of traditional waveguides. SIWs can be integrated into a planar substrate while still providing low loss, high power handling capability similar to conventional 3D waveguides. Consequently, SIWs have emerged as an important solution for millimeter-wave transmission lines.

For the fabrication of SIWs in millimeter-wave applications, selecting substrates with low dielectric loss (tanδ) and high-precision machineability is crucial for achieving low-loss signal transmission. Substrates used in these applications must also support the formation of fine-pitch patterns. Conventional low-loss dielectric substrates, such as printed circuit boards (PCBs), low-temperature co-fired ceramics (LTCCs) [11], and organic substrates like liquid crystal polymers (LCPs) [12], have been extensively utilized. However, these materials often face challenges in achieving micrometer-scale pattern precision and tolerances, which are essential for millimeter-wave components. To address these issues, glass substrates have been explored as a promising alternative due to their excellent electrical performance, ability to form fine features and vias, robust mechanical stability, and compatibility with cost-effective, large-panel processing. To implement the SIW structure on glass substrates, through glass via (TGV) technology is employed [13,14,15,16,17,18,19,20,21,22,23,24,25,26,27]. Typically, empty TGV holes are created using techniques such as laser or ultrasonic drilling, powder blasting, or wet chemical etching, followed by filling with conductive metals using sputtering or electro/electroless plating techniques. However, each of these methods has its own limitations such as, for example, long processing time, higher costs, difficulties in wafer-level process, etc. Specifically, it is a challenging issue to completely fill up the micrometer-scale TGV holes with conductive materials without any voids [28]. Recent electroplating approaches tend to use partial filling conformal or super-conformal electroplating widely instead of bottom-up electroplating due to the time consumption and cost [29,30], but they cannot be easily applied to many TGVs with relatively rough TGV-hole sidewalls. In conductive paste filling methods, metallic pastes such as nano-silver or conductive copper paste are filled into TGV holes by utilizing screen printing and sintering or 3D-printing techniques [31,32]. A vacuum environment can prevent void formation, but the volume reduction after sintering is a potential issue. In addition, an interesting approach using magnetic self-assembly of metal (nickel) wires into the TGV holes was reported [33]. This method provides high density and a high aspect ratio metallization of TGVs for various wall morphologies, but multiple magnets are required for magnetic control.

In this paper, we propose a method for fabricating glass substrates with conductive tin (Sn) TGVs by vacuum suctioning of molten tin into the TGV holes and apply this approach to fabricate SIWs operating at millimeter-wave frequencies. Tin is a well-known soft metal with excellent corrosion resistance, malleability, and good thermal/electrical conductivity that has been widely used in electronic manufacturing industries [34]. Due to its low melting point and good electrical conductivity, tin—often alloyed with lead or other metals—is essential in soldering, which provides conductive connections in electronic components. In our work, TGV holes are formed by reflowing glass into deep etched cavities within a vacuum-bonded silicon carrier wafer, followed by polishing and successive deep etching of exposed silicon vias embedded in the glass. Compared to the individual laser or ultrasonic drilling, this process enables wafer-level manufacturing of precise TGV holes. These holes are then filled with molten tin using a custom-designed vacuum suctioning device controlled by a solenoid valve linked to a microcontroller unit. Unlike other via-filling techniques such as electroplating or sputtering, this technique significantly reduces the total processing time for fully filling the vias to just a few minutes while still minimizing the risk of void formation due to the liquid properties of molten tin. We designed and fabricated an SIW incorporating tin TGVs using the proposed process and measured its performances from 20 to 35 GHz, demonstrating its potential applicability in millimeter-wave applications.

## 2. Design and Simulation

**Figure 1:**
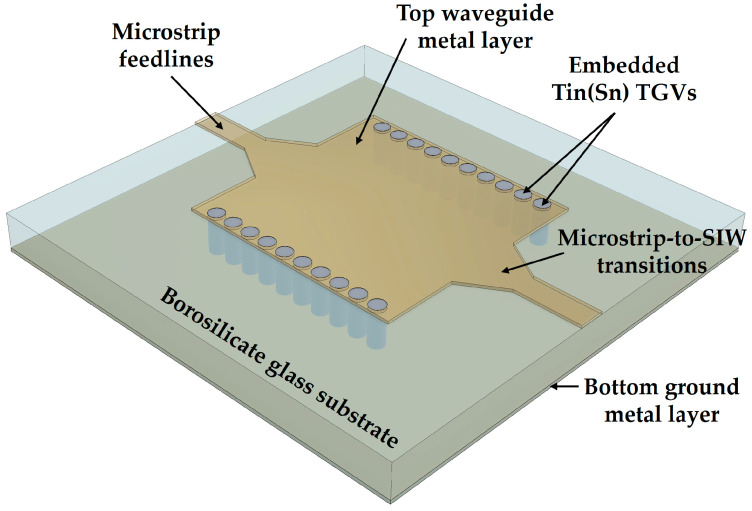
Conceptual 3D view of the proposed SIW with tin TGVs.

Figure 1 illustrates the conceptual 3D view of the proposed SIW with tin TGVs. The SIW is composed of a glass substrate, top metal layers including feedlines and transitions, a bottom ground metal layer, and tin TGV arrays. A borosilicate glass substrate with TGV holes fabricated by a MEMS-based glass reflow process is used as a dielectric substrate of the SIW. Tin TGVs filling the empty TGV holes electrically connect the top metal layer of the SIW to the bottom ground plane, serving as the sidewalls of a classical rectangular waveguide. To feed the SIW, a 50 Ω matched microstrip line is formed with a tapered transition part providing smooth field transition from quasi-TEM mode of the microstrip line to the TE_10_ mode of the SIW over a broad bandwidth. The thickness of the SIW substrate was preset to 350 μm considering the mechanical stability of the wafer during the whole fabrication processes.

**Figure 2:**
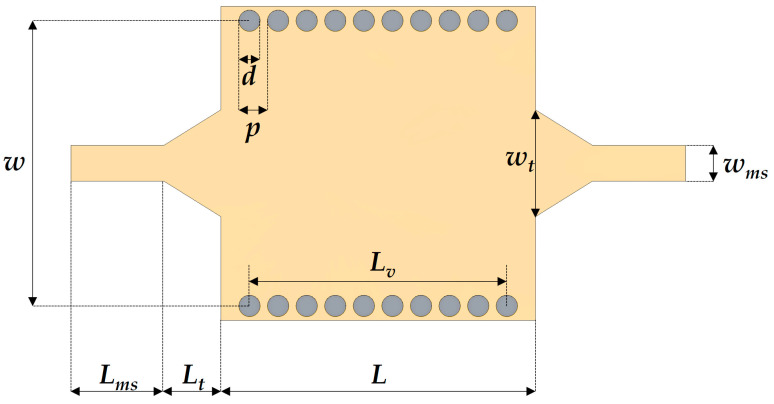
Top view of the SIW with design parameters.

The dimensions of the top metal patterns and tin TGVs are important design parameters to determine the frequency characteristics of the SIW. Figure 2 illustrates the top view of the SIW with its design parameters. The cutoff frequency of the SIW operating in the dominant TE_10_ mode, $f_{c,\;10}$, is determined by the well-established equation [35]:

(1) $$f_{c,\;10}=\frac{c}{2w_{eff}\sqrt{\varepsilon_{r}}}$$

where c is the speed of light, $\varepsilon_{r}$ is the relative permittivity of the dielectric substrate, and $w_{eff}$ is the effective width of the SIW. The effective width of the SIW accounts for the effects of via arrays, which replace the continuous sidewalls of conventional waveguides. An empirical expression for $w_{eff}$ is provided as follows [36]:

(2) $$w_{eff}=w-1.08\frac{d^{2}}{p}+0.1\frac{d^{2}}{w}\;\;\;\;\;$$

where w is the center-to-center distance between the two via rows, d is the diameter of a via, and p is the pitch between the vias. Equation (2) offers high accuracy for conditions where p/d<3 and d/w<0.2 [36]. In addition, for ensuring reasonably small radiation losses due to energy leakage through gaps between vias, the condition p/d<2.5 should be satisfied [3].

Following the design guidelines, an SIW with a TE_10_-mode cutoff frequency of 18.6 GHz, calculated from Equation (2), was developed. The frequency characteristics of the designed SIW were analyzed using the finite element method (FEM) with commercial full-wave electromagnetic simulation software (Ansys HFSS, Ansys Electronics Desktop 2024 R2, Ansys, Inc., Canonsburg, PA, USA). For FEM simulation, 50-Ω waveguide ports were set for the input/output ports, and the radiation box was defined as an air material containing the SIW specimen. Metal layers were set to have a finite conductivity, given below. Analysis was performed from 10 to 35 GHz using an interpolating sweep. The glass substrate used in the design was a Borofloat-33 glass wafer (SCHOTT AG, Mainz, Germany) with a dielectric constant of 4.6 and a loss tangent of 0.0037. The conductivity of the tin TGVs was set to be σ = 8.7 × 10^6^ S/m. For comparison, SIWs with identical dimensions but incorporating conventional copper TGVs (σ = 5.8 × 10^7^ S/m) and low-resistive silicon TGVs (σ = 5.0 × 10^4^ S/m) were also simulated. The dimensions of the designed SIW were as follows: *d* = 0.3 mm, *p* = 0.4 mm, *w_ms_* = 0.5 mm, *L_ms_* = 1.3 mm, *w_t_* = 1.5 mm, *L_t_* = 0.8 mm, *L* = 4.4 mm, *L_v_* = 3.9 mm, and *w* = 4.0 mm.

**Figure 3:**
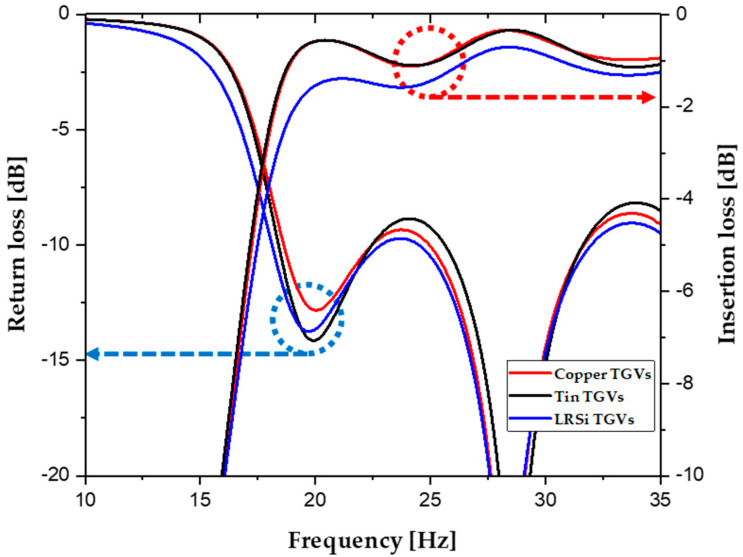
Simulated *S*-parameters of the SIWs with copper, tin, and low-resistive silicon TGVs.

The HFSS simulations were conducted to obtain frequency characteristics over the range of 10 to 35 GHz, and the resulting *S*-parameters are shown in Figure 3. The left *y*-axis denotes the return loss, while the right *y*-axis indicates the insertion loss. From 20 to 35 GHz, the SIW with tin TGVs exhibited an average insertion loss of 0.76 ± 0.18 dB, closely matching the 0.74 ± 0.16 dB of the SIW with copper TGVs. In comparison, the SIW with pure low-resistive silicon TGVs demonstrated a significantly higher average insertion loss of 1.2 ± 0.17 dB. Despite the conductivity of tin being an order of magnitude lower than that of copper, the SIW with tin TGVs achieved comparable insertion loss values, with only a slight increase observed above 30 GHz. As expected, the SIW with tin TGVs outperformed the SIW with silicon TGVs in terms of electrical performance.

## 3. Fabrication Process and Result

**Figure 4:**
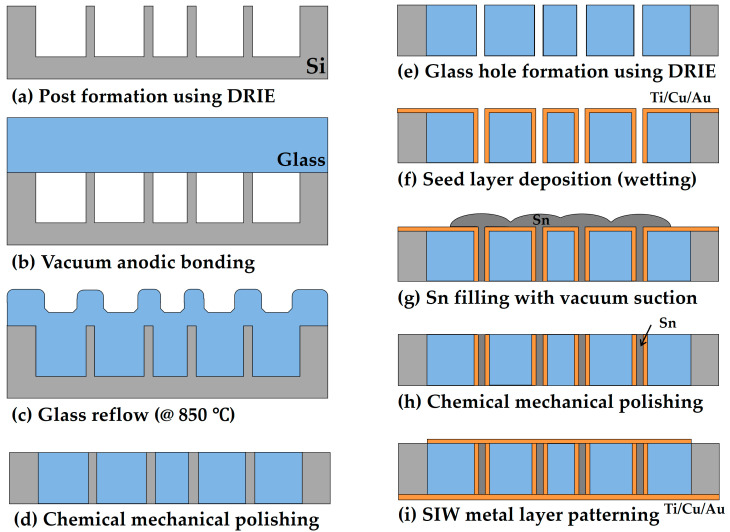
Overall fabrication process of the proposed SIW with tin TGVs.

The overall fabrication process for the proposed SIW is based on micromachining processes. Figure 4 illustrates the process flow of the SIW with tin TGVs. The process begins with deep reactive ion etching (DRIE) of a 4-inch silicon wafer with a thickness of 0.5 mm to form cavities that accommodate the melted glass during the glass reflow process (Figure 4a). To achieve the necessary etch selectivity for deep etching of more than 400 µm, a 12.1-µm-thick AZ P4620 photoresist layer was patterned onto the wafer using photolithography, serving as the etch mask for DRIE. The silicon wafer, etched to a depth of approximately 420 µm, was bonded with a Borofloat-33 glass wafer using anodic bonding (EVG 501 anodic bonder, EV Group, St. Florian am Inn, Austria) (Figure 4b). Since anodic bonding was performed in a vacuum chamber at 1.5 × 10^−4^ Torr, the cavity formed between the silicon and glass also remained under vacuum. The bonded wafer was subsequently heated in an atmospheric furnace at 850 °C. During this stage, the pressure difference between the atmospheric pressure and the vacuum inside the sealed cavity caused the glass to reflow into the silicon cavities (Figure 4c). After cooling, chemical mechanical polishing (CMP) was applied to create a glass substrate embedded with silicon TGVs (Figure 4d). The silicon via region of the fabricated substrate was then removed by DRIE to produce empty TGV holes (Figure 4e).

**Figure 5:**
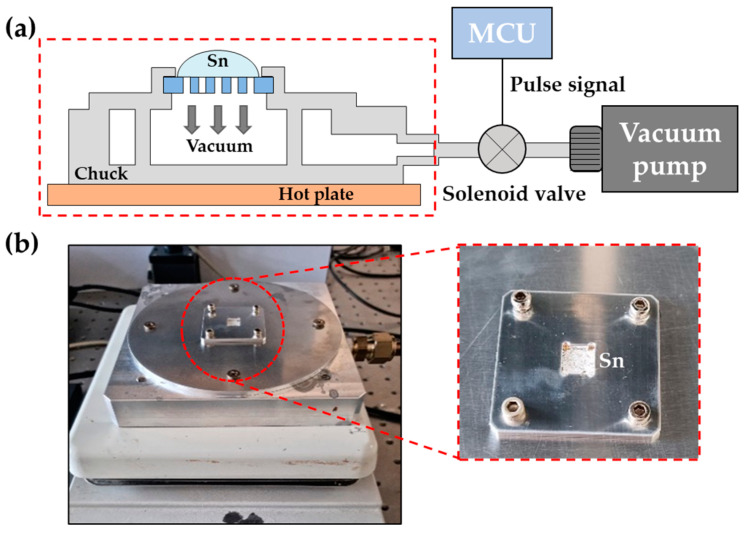
Vacuum suctioning system for fabricating tin TGVs: (**a**) Schematic view of the vacuum suctioning system. (**b**) Image of the vacuum suction chuck.

To fill up the TGV holes with tin, a wetting layer of Ti/Cu/Au (10/200/100 nm) was first deposited using a sputtering process (Figure 4f). This wetting layer is essential for retaining the molten tin within the via holes during the suction process. A specially designed vacuum suctioning device was employed to draw molten tin into the TGV holes, as depicted in Figure 5a. The process begins with securing the wafer or specimen onto an aluminum vacuum chuck, which is then heated on a hot plate (Figure 5b). Tin pellets are placed on the specimen and heated to 250 °C until melted. A solenoid valve, controlled by a microcontroller unit (MCU), is positioned between the vacuum pump and the vacuum chuck. When the MCU activates the pulse signal, the solenoid valve opens, initiating the suction process. In the experiment, the driving pulse was applied for 200 ms, followed by natural exhaust at the atmospheric pressure for 800 ms. The pressure inside the vacuum chuck was monitored by an analog pressure sensor connected to it. The vacuum suction was performed at a pressure of 600 Torr with a frequency of 1 Hz and a 20% duty cycle. The empty TGV holes in the glass were completely filled with molten tin by just 1 min of suctioning (Figure 4g). This method significantly reduces the time required to completely fill up the high-aspect-ratio TGV holes with conductive material. After natural cooling, the overfilled tin was polished (Figure 4h). Then, the top waveguide metal pattern composed of Ti/Cu/Au (10/300/100 nm) was formed on the glass substrate with tin TGVs. The bottom side of the substrate was fully covered with a metal layer of the same thickness as the top metal pattern (Figure 4i).

**Figure 6:**
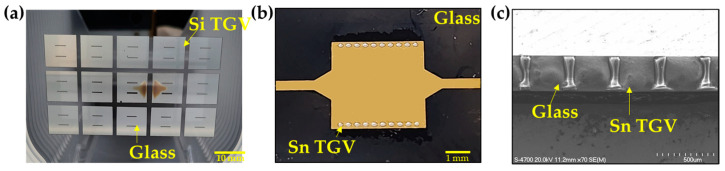
Fabrication results: (**a**) Image of the wafer after chemical mechanical processing of the glass-reflowed substrate. Silicon TGVs are embedded in the reflowed glass. (**b**) Image of the fabricated SIW with tin TGVs. (**c**) FE-SEM cross-sectional image of the SIW with tin TGVs. The tin is filled into the TGV holes in the glass without voids.

Figure 6 presents the results of the fabrication process. Figure 6a shows an image of the wafer taken after CMP of the glass-reflowed substrate (Figure 4d), illustrating that the silicon TGVs are accurately embedded in the glass at their designed locations. Figure 6b displays the image of the fabricated SIW with tin TGVs after all fabrication steps have been finished. To confirm the absence of voids in the tin-filled TGVs, a cross-sectional image of the specimen was captured using a field-emission scanning electron microscope (FE-SEM). Figure 6c verifies that the tin TGVs are completely filled with no voids present.

## 4. Experimental Results and Discussion

Before measuring the RF performances of the fabricated SIW, the dimensional parameters of the specimen were measured and compared with the designed values, as shown in Table 1. The dimensions of the top metal patterns, including microstrip lines and transitions of the SIW, which were determined by photolithographic process, showed relatively small variations. The largest deviation was observed in the diameter of the vias, which is related to the initial DRIE process used to form the cavities in the silicon wafer (Figure 4a). The diameter of the tin via (*d*) was found to be approximately 60 μm smaller than the designed value. This indicates that the diameter of the silicon post structures for the TGV holes was reduced during the first DRIE step. This reduction is primarily due to the faster erosion of the photoresist etch mask at the edges of the silicon posts compared to the center part during the extended DRIE process. Since dimensional deviations can influence the RF characteristics of millimeter-wave devices, where high dimensional accuracy is required, an EM simulation was conducted again using these measured parameters.

**Figure 7:**
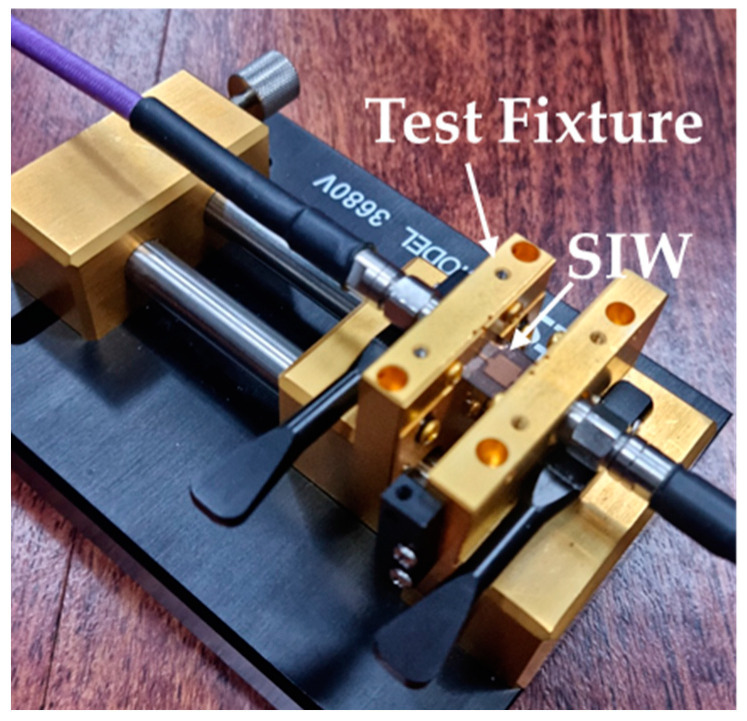
Experimental setup for measuring *S*-parameters of the fabricated SIW with tin TGVs.

The frequency characteristics of the fabricated SIW were measured using a commercial test fixture (3680V, Anritsu Corp., Atsugi, Japan) and a vector network analyzer (N5227B, Keysight Technologies, Santa Rosa, CA, USA). Figure 7 shows the SIW mounted on the test fixture. A standard short-open-load-thru (SOLT) calibration process was performed using a commercial calibration kit (36804B-10M, Anritsu Corp., Japan).

**Figure 8:**
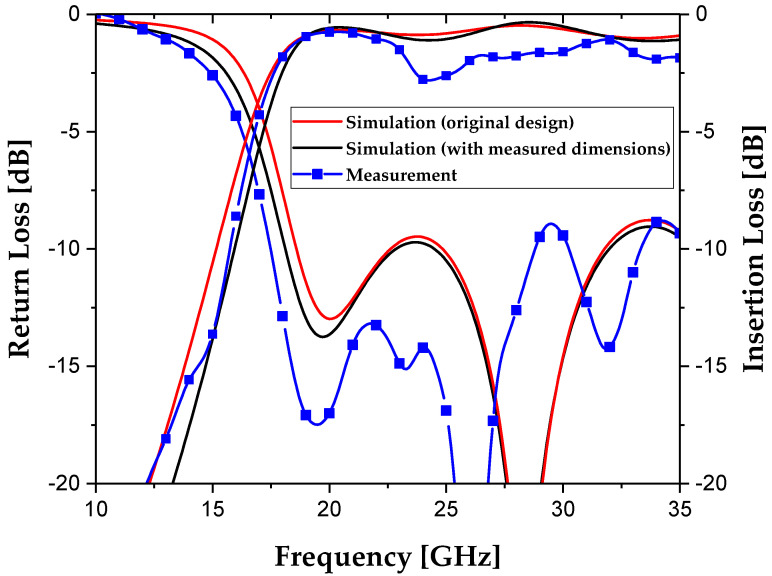
Measured *S*-parameters of the fabricated SIW with tin TGVs compared with the simulation results.

The fabricated SIW with tin TGVs was connected to the test fixture, and its *S*-parameters were measured over the frequency range from 10 to 35 GHz. Figure 8 shows the measured *S*-parameters of the fabricated SIW, compared with two simulation results. The 3-dB cutoff frequency from the minimum insertion loss was measured to be 17.1 GHz for the fabricated SIW, which is close to the simulation result of 17.5 GHz based on the measured dimensions. The measured insertion loss was expressed as an average value with a standard deviation over the frequency range from 20 to 35 GHz. The average insertion loss of the fabricated SIW with tin TGVs was measured to be 1.64 ± 0.54 dB, which is considerably higher than the simulation result of 0.79 ± 0.27 dB. This discrepancy is mainly attributed to unexpected large deviation in the 24–26 GHz region. As shown in Figure 8, no significant discrepancies were observed between the two simulation results, except that the average insertion loss in the simulation using measured dimensions slightly increased from 0.76 ± 0.18 dB to 0.79 ± 0.27 dB. This indicates that the dimensional change in via diameter due to fabrication errors in the DRIE process does not significantly affect the overall performance of the SIW. Therefore, it is suggested that other factors, such as a decrease in conductivity of the tin vias, an increase in loss tangent value for the glass substrate at higher frequencies, roughened surfaces of the substrate during the DRIE process to remove silicon via structures for TGV hole forming, or issues in the experimental measurement setup, likely contributed more to the increased insertion losses than the fabrication errors.

In Table 2, performances of the fabricated SIW in this work were compared with those of other SIWs demonstrated on borosilicate-glass-based substrate around similar frequency ranges, including our previous results. Compared to our previous SIWs with pure copper TGVs [18] and tungsten-coated silicon TGVs [22], the SIW with tin TGVs in this work showed a much higher insertion loss, at least more than 0.5 dB. Since the same electrical parameters of the glass substrate were used and the dimensional lengths of all SIWs showed no significant differences, the primary cause of this higher insertion loss is presumed to be that the real conductivity of the fabricated tin vias is much lower than the assumed value. This conjecture was indirectly verified by measuring electrical resistance of tin vias. The tin via array with a diameter of 150 μm was fabricated separately, and the electrical resistance of 20 randomly-selected vias was measured using the 4-probe method. When using the theoretical conductivity of tin (σ = 8.7 × 10^6^ S/m), the resistance of a single tin via was calculated to be about 2.2 mΩ. However, the actual resistance was measured to be about 69.8 mΩ, which is about 30 times higher than the theoretical value. In addition, the measured resistance includes the interface resistance between the Ti/Cu electrode, which could be increased further due to the oxidation of copper and tin surfaces during the process, especially at higher processing temperatures. Obtaining low-temperature conditions for vacuum suction of molten tin would be helpful to further reduce the insertion losses.

## 5. Conclusions

In this study, we proposed a method to fabricate tin TGV structures in the glass substrate by filling the TGV holes with molten tin using a vacuum suctioning system. The TGV holes in the glass wafer were created through wafer-level micromachining processes, which involve reflowing the glass into the etched cavity in a vacuum-bonded silicon carrier wafer. Following the removal of the silicon TGVs embedded in the glass substrate, molten tin was introduced into the TGV holes using a custom-designed vacuum suction device. The TGV holes were completely filled with molten tin, without any voids, in only a minute. This TGV-forming process was applied to fabricate an SIW operating in the 20 to 35 GHz range. Over this frequency range, the fabricated SIW demonstrated a measured insertion loss of 1.64 ± 0.54 dB, including all feedlines and transitions. The proposed method offers a very fast production of conductive TGVs compared to the traditional techniques, highlighting its potential for cost-effective and time-efficient fabrication of millimeter-wave passive components with via structures in glass.

## Figures and Tables

**Table 1:** Comparison of the dimensional parameters for the fabricated SIW.

Parameters	Designed	Measured	Description
*d*	0.3 mm	0.239 mm	Diameter of a single TGV
*p*	0.4 mm	0.398 mm	Pitch between the TGVs
*w_ms_*	0.5 mm	0.494 mm	Width of the microstrip line
*L_ms_*	1.3 mm	1.292 mm	Length of the microstrip line
*w_t_*	1.5 mm	1.506 mm	Width of the tapered transition
*L_t_*	0.8 mm	0.803 mm	Length of the tapered transition
*L*	4.4 mm	4.375 mm	Length of the SIW
*L_v_*	3.9 mm	3.797 mm	Center-to-center distance between the two end vias
*w*	4.0 mm	3.981 mm	Center-to-center distance between the two via rows
*h*	0.35 mm	0.351 mm	Thickness of the substrate

**Table 2:** Performance comparison of the SIWs fabricated on the borosilicate-glass-based substrate.

Ref.	Substrate $(\mathrm{Thickness}/\varepsilon_{r}$/tan δ)	TGV Material	Frequency [GHz]	Device Length [mm]	Insertion Loss [dB]
[18]	Borosilicate glass (350 μm/4.6/0.0037 ^1^)	Electroplated Cu	20–45	10.0 ^2^	<0.95 dB ^2^
[22]	Borosilicate glass (350 μm/4.6/0.0037 ^1^)	Tungsten-coated Si	20–45	7.0 ^2^	<1.15 dB ^2^
[23]	Borosilicate glass with low-loss polymer lamination (100 μm/5.4/0.006)	Electroless-plated Cu+ semi-additive patterning (SAP) metallization	24–40	4.0 ^3^	0.64 dB ^3,4^
This work	Borosilicate glass (350 μm/4.6/0.0037 ^1^)	Vacuum suctioned Tin (Sn)	20–35	8.6 ^2^	1.64 ± 0.54 dB ^2,5^

^1^ Values @ 1 MHz. ^2^ Values including feedlines and transitions. ^3^ Values of only waveguide section. ^4^ De-embedded value. ^5^ Averaged value.

## Data Availability

The original contributions presented in this study are included in the article. Further inquiries can be directed to the corresponding author(s).
